# The lncRNA NEAT1 Inhibits miRNA-216b and Promotes Colorectal Cancer Progression by Indirectly Activating YY1

**DOI:** 10.1155/2022/8130132

**Published:** 2022-10-10

**Authors:** Yuping Zhu, Xuanying Wang, Linfeng Zheng, Dechuan Li, Zhuo Liu, Lisong Teng

**Affiliations:** ^1^Zhejiang University, Hangzhou 310058, China; ^2^Department of Colorectal Surgery, Cancer Hospital of the University of Chinese Academy of Sciences CHUCAS, Zhejiang Cancer Hospital, Hangzhou 310022, China; ^3^Department of Integrated Chinese and Western Medicine, Cancer Hospital of the University of Chinese Academy of Sciences CHUCAS, Zhejiang Cancer Hospital, Hangzhou 310022, China; ^4^Department of Pathology, Cancer Hospital, University of Chinese Academy of Sciences CHUCAS, Zhejiang Cancer Hospital, Hangzhou 310022, China; ^5^Department of Surgery, First Affiliated Hospital of Zhejiang University, Hangzhou 310003, China

## Abstract

**Background:**

*Nuclear Paraspeckle Assembly Transcript 1* (NEAT1) is commonly considered an oncogene in various cancers. The long noncoding RNA NEAT1 has been reported to be overexpressed in colorectal cancer (CRC). However, the exact role of NEAT1 in CRC remains unknown. Our research aimed to explore the function of NEAT1 in the tumorigenesis and the development of CRC.

**Methods:**

Real-time quantitative PCR (qRT-PCR) was used to detect the NEAT1, miR-216b, and YIN-YANG-1 (YY1) mRNA levels in CRC tissues and cells, then immunohistochemistry (IHC) was used to detect the expression of YY1 in CRC tissues. Luciferase reporter, qPCR, western blot, and DNA pulldown assays were conducted to study the relationships between NEAT1, miR-216b, and YY1. Flow cytometry analysis was performed for cell cycle and apoptosis analyses, and a colony formation assay was performed to test cell proliferation. Transwell assays were performed to detect cell invasion and migration.

**Results:**

The NEAT1 expression was significantly upregulated in CRC tissues compared with its expression in normal tissues, and downregulation of NEAT1 suppressed the proliferation, migration, and invasion of CRC cells. Moreover, we found NEAT1 decreased the miR-216b level directly, and the suppression of miR-216b could inhibit the function of downstream YY1. However, overexpression of YY1 accelerated CRC cell proliferation, migration, and invasion.

**Conclusion:**

Our results indicated that NEAT1 acted as an oncogene in CRC and promoted the progression of CRC by directly sponging miR-216 b expression to activate the expression of YY1. The NEAT1/miR-216b/YY1 axis may be a novel therapeutic target for CRC.

## 1. Introduction

Colorectal cancer (CRC), including colon cancer and rectal cancer, is one of the most common malignancies in the world, which has threatened the global health [1]. Although the existing methods of diagnosis and treatment continue to improve, it also exhibits the low rate of 5-year overall survival and poor prognosis [2]. Due to its complex process of pathogenesis involving many factors, the clear mechanism of CRC still retain unknown, which motivate the clinical treatment to explore the CRC progression.

Increasing attention has focused on the effect of long noncoding RNAs (LncRNAs), a subfamily of noncoding RNAs, in regulating the CRC progress [3]. Emerging evidence has shown that lncRNAs are important in the occurrence and progression of cancers by regulating cell cycle progression, apoptosis, and metastasis [4]. PANDAR, HOTAIR, HULC, H19, MALAT1, XIST, and NEAT1 have been considered as the oncogenes in various cancers, which can act as a “sponge” and compete for the binding of miRNAs of other genes [5–10]. LncRNA NEAT1 (NEAT1), encoding two variants of Neat1_v1 (3.7kb) and Neat1_v2 (23kb), is transcribed from a gene locus called multiple endocrine tumor type 1 in familial tumor syndrome on chromosome 11, which abnormally expressed in many malignant tumors, including CRC [11]. Nevertheless, the underlying molecular mechanism of NEAT1 needs further elucidation. Moreover, NEAT1 could sponge microRNAs such as miR-150-5p, miR-193a, miR-205-5p, and miR-34a to regulate CRC progression, leading to downstream signal transduction to induce chemotherapy resistance or metastasis [12–15].

In our research, we detected the expression of NEAT1 in human CRC tissues and cells. Subsequently, functional experiments were prepared to verify the carcinogenic effect of NEAT1. In mechanism, the regulatory relationship between NEAT1 and micRNA-216b was further studied which has not reported previously. Notably, we provided evidence that NEAT1 directly sponged the expression of miR-216b, which consequently removed functional regulation of the downstream YY1. This study provides new insights into clinical treatment and further intervention targets for CRC.

## 2. Materials and Methods

### 2.1. Patient Specimens

All 57 paired CRC tissues and normal tissues stored in biobank were obtained from those diagnosed CRC patients who underwent no preoperative therapy prior to surgical resection at Zhejiang *Cancer* Hospital (Hangzhou, China) from 2013 to 2017. Tissue collection and manipulation were authorized by the Ethics Committee of Zhejiang *Cancer* Hospital (ethics number: IRB-2020-26). Informed consents were collected from all subjects.

The specimens were frozen in liquid nitrogen after surgery and kept at −80°C. The detailed clinical and pathological data from these 57 CRC specimens are shown in Table 1. The overall survival time and time from diagnosis to death were recorded to detect the prognostic status. Conventional receiver operating characteristic (ROC) curve analysis was used to confirm the cut-off value of NEAT1 expression. Furthermore, Youden index, which consisted of specificity and sensitivity, was prepared to help calculate the cut-off of the expression level (specificity%+sensitivity%−100%), the largest one of which was the cut-off value.

### 2.2. Quantitative Real-Time Polymerase Chain Reaction (qRT-PCR)

Total RNA was isolated from the paired tissues using the Trizol reagent (number: 15596–018, Invitrogen, Carlsbad, USA) according to the standard protocol in the kit. After reverse transcription of total RNA by using a cDNA reverse transcriptase kit (Takara, code number: 6215A, purchased from ElifeBio, Hangzhou, China), we obtained the cDNA. The expression levels of LncRNA NEAT1 and miR-216b were measured using Applied Biosystems 7500 Real Time PCR System with a Green One-Step qRT-PCR SuperMix Kit (Transgen, code number: AQ211-01, Beijing, China). The reaction conditions were as follows: predenaturation at 95°C for 5 min, denaturation at 95°C for 10 s, and annealing at 60°C for 30 s for 35 cycles. The results were normalized to the expression levels of GAPDH or U6 snRNA using the 2-ΔΔCT method for quantification. Primers were devised and produced by Sangon Biotech (Shanghai, China), and the sequences of PCR primers are shown in Table 2.

### 2.3. Western Blot Assays

The details of western blot assays were performed as described in our previous published research [10]. Fresh tumor tissues and normal tissues treated with liquid nitrogen were crushed and then lysed in RIPA lysis buffer supplemented with proteinase inhibitor cocktail (Roche, number: 5892970001). In brief, lysed proteins were isolated by SDS-PAGE, transferred to PVDF membranes, and incubated with the following primary antibodies: anti-GAPDH antibody (1 : 3000 dilution, rabbit, Abcam, ab9485) andanti-YY1 antibody (1 : 500 dilution, rabbit, Abcam, ab109228). Following incubation with the appropriate HRP-conjugated secondary antibodies, the bands were visualized using Pierce™ ECL western blotting substrate (Thermo Scientific, number: 32106, USA). The signal intensity of the protein was further determined by ImageJ software (Madison, WI, USA).

### 2.4. Immunohistochemistry (IHC)

The detail of the assay referred to the previous study [16]. After cut into 4-*µ*m sections all the tissues were deparaffinized and treated with EDTA (pH 9.0) to antigen retrieval in a microwave for 20 min. Then, Autostainer Link 48 machine (Dako, Denmark A/S, Denmark) was performed for staining. Subsequently, primary anti-YY1 antibody (1 : 100, Abcam, ab109228) was added to the sections as well as their coordinate secondary antibody, while PBS buffer was used as a blank control instead of the antibody. EnVision Flex Kit (K802321–2CN) was used as the second antibody (Dako, Denmark A/S, Denmark). Percentage of positively stained cells and staining scores were used to assess the IHC results and the detail is shown in Table S1.

### 2.5. Cell Culture and Transfection

All cell lines (LoVo, Caco-2, SW620, HT29, HCT-116, and the normal colon cell line NCM460) were purchased from ATCC and cultured in RPMI 1640 medium (RPMI 1640; Gibco, Grand Island, NY, USA) containing 10% fetal bovine serum (FBS; Gibco, Grand Island, NY, USA) and 1% penicillin/streptomycin (ElifeBio, Hangzhou, China) with 5% CO_2_ at 37°C. HCT-116 and SW620 cells (1 × 105 cells) were seeded in 6-well plates in RPMI 1640 medium for 12 h. Then, the cells were treated with NEAT1-or YY1-specific shRNA as well as the negative control with 0.2% Lipofectamine 3000 in RPMI 1640 medium. The plasmids YY1 and NEAT1 were purchased from ElifeBio (Hangzhou, China). The miR-216 b inhibitor, miR-216 b mimics, and mimic normal control were purchased from ElifeBio (Hangzhou, China). The detailed sequences are listed in Table 2. After 5 h, the transfection reagent was replaced with RPMI 1640 medium. Forty-eight hours later, the cells were collected for further experiments.

### 2.6. Cell Proliferation Analysis

Cell proliferation or viability was determined by a CCK-8 assay (HY–K0301, MCE). HCT-116 and SW620 cells (1 × 10^5^ cells/well) transfected with or without the plasmid were seeded in 96-well plates. In brief, on the day of measuring the growth rate of the treated cells, 100 *µ*l of spent medium was replaced with an equal volume of fresh medium containing 10% CCK-8 reagent. Cells were incubated at 37°C for 1 h, and the absorbance was subsequently detected at 450 nm with a microplate reader.

### 2.7. Cell Colony Analysis

HCT116 and SW6200 cells, transfected with plasmid or not, were seeded in 6-well plates in RPMI1640 medium at 37°C, half of the medium was replenished on day 5 and the medium was discarded on day 14. Cells were washed with phosphate buffer saline (PBS), fixed with methanol for 10 min, and stained with crystal violet for 5 minutes, and then observed under a microscope. Cells in five fields were selected for counting.

### 2.8. Cell Invasion and Migration Assays

Cell invasion and migration were tested by a transwell chamber with Matrigel (8-*μ*m pore size; BD Biosciences, USA) according to the manufacturer's protocol. The detail referred to the previous study [12].

### 2.9. Flow Cytometry Cell Cycle and Apoptosis Analysis

Flow cytometry assay for cell cycle and apoptosis was prepared referring to a previous article [17].

### 2.10. Pull-Down Assay with Biotinylated NEAT1 DNA Probe

The DNA fragment containing the full-length NEAT1 sequence or negative control sequence was PCR amplified by a T7-containing primer and then bound to GV394 (Invitrogen, Shanghai, China). The obtained plasmid DNAs were digested to a linear form by the restriction enzyme XhoI. Biotin-labeled RNAs were conversely transcribed, and then qRT-PCR was used to analyze target the RNA expression according to the method described previously [18].

### 2.11. Statistical Analysis

All experimental data were analyzed by GraphPad Prism 8.0, and the results are shown as the mean ± SD (standard deviation). Chi-square tests were performed to detect the correlation between the NEAT1 expression and clinicopathological factors. Student's *t*-test was performed to compare the differences between the two groups. Comparisons among multiple groups were made by one-way ANOVA. Survival analysis was performed via the Kaplan–Meier method accompanied by Cox regression analysis for univariate and multivariate analyses. *P* < 0.05 was considered significant.

## 3. Results

### 3.1. Detecting NEAT1 from TCGA in Cancers by Online Tools

**Figure 1:**
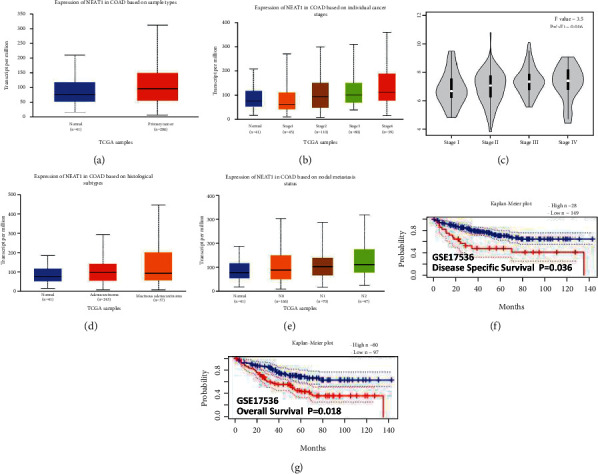
Detecting NEAT1 from TCGA in cancers by online tools (a) the expression of NEAT1 was analyzed by “UALCAN” and the data were obtained from TCGA. NEAT1 overexpressed in colon cancer tissue (*n* = 286) and the paired normal colon tissue (*n* = 41). (b) NEAT1 was further analyzed by “UALCAN” in different clinical stages and overexpressed higher in stage IV and stage III than in stage I and stage II. (c) NEAT1 was analyzed by “Gepia” in different clinical stages and found overexpressed higher in stage IV and stage III than in stage I and stage II. (d) The expression of NEAT1 in different pathological pattern by “UALCAN,” and NEAT1 overexpressed higher in mucinous adenocarcinoma than that in adenocarcinoma or normal. (e) The expression of NEAT1 in different nodal stages by “UALCAN,” and NEAT1 overexpressed higher in N2 and N1 stages than that in N0 and N1 stages. (f) and (g) NEAT1 overexpression suggested the lower disease -ree survival and overall survival analyzed by “PrognoScan” ^*∗*^*P* < 0.05. ^*∗∗*^and *p* < 0.01.

**Figure 2:**
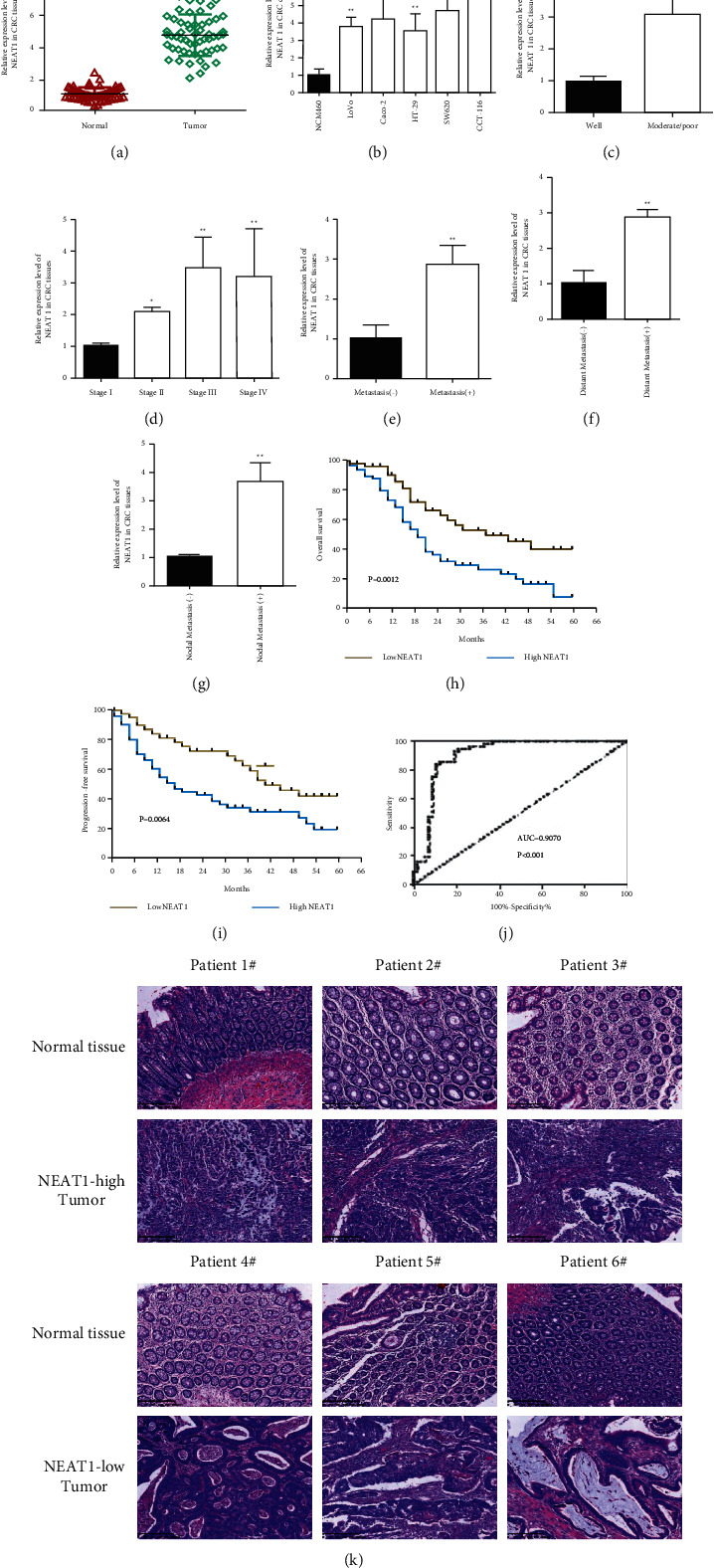
Upregulation of NEAT1 in the CRC tissues and cells. (a) NEAT1 is overexpressed in the CRC tissues (*n* = 32) compared to the normal tissues (*n* = 32) by qRT-PCR. (b) Detecting NEAT1 expression in CRC cells compared to normal cells by qRT-PCR. (c) Exploring NEAT1 expression in different histological differentiation (well: 25 and poor: 32) of CRC tissues by qRT-PCR. (d) Discovering NEAT1 expression in different clinical stages (stage (i): 25, stage II: 21, stage III: 24, and stage IV: 31) of CRC tissues by qRT-PCR. (e) NEAT1 detection in CRC with metastasis or nonmetastasis tissues (positive: 64 and negative: 79) by qRT-PCR. (f) Upregulation of NEAT1 in CRC with distant metastasis tissues (positive: 19 and negative: 21) determined by qRT-PCR. (g) Upregulation of NEAT1 in CRC with distant metastasis tissues (positive: 13 and negative: 11) detected by qRT-PCR. (h) (i) The Kaplan–Meier survival curve demonstrated that upregulation of NEAT1 in patients with CRC indicated poor prognosis in the overall survival (H) and progress in-free survival (I). ^*∗*^*P* < 0.05 and ^*∗∗*^*p* < 0.01. (j) ROC curve analysis of the NEAT1 expression in colorectal tissues. (k) Different expressions of NEAT1 is related to different histological differentiation, shown by HE staining.

According to the public, database “UALCAN” (http://ualcan.path.uab.edu) from the *Cancer* Genome Atlas (TCGA) in cancers, we found NEAT1 overexpressed in tumor tissues (Figure 1(a), *p* < 0.01) as well as upregulated higher in stage IV and stage III than that in stage I and stage II (Figure 1(b), *p* < 0.01), which was also identified in “Gepia” database (http://gepia.cancer-pku.cn/) (Figure 1(c), *p* < 0.01). NEAT1 showed much higher expression in mucinous adenocarcinoma than adenocarcinoma(Figure 1(d), *p* < 0.01). Higher level expression of NEAT1 also indicated the severe lymph node metastasis stage (Figure 1(e), *p* < 0.01) as well as poor disease-specific survival (Figure 1(f), *p*=0.036) and overall survival (Figure 1(g), *p*=0.018) in cohort GSE17536. Referred to the results from the public database, we have tested the NEAT1 expression in CRC tissues and cell lines that its actually overexpressed in CRC tissues (Figure 2(a), *p* < 0.01) and cells (Figure 2(b), *p* < 0.01) compared with normal. It was similar with the database result that NEAT1 showed the high expression in poorly differentiated tissues not the well ones (Figures 2(c) and 2(k), *p* < 0.01). Moreover, we found NEAT1 overexpressed in CRC patients with the stage III/IV compared with the stage I/II (Figure 2(d), *P* < 0.05). Furthermore, CRC patients with metastasis especially nodal metastasis and liver metastasis exhibited a higher expression level of NEAT1 than nonmetastasis ones (Figures 2(e)–2(g), *p* < 0.01). In order to identify the association of NEAT1 in prognosis, we explored the cut-off value of NEAT1 in our CRC specimen, the result of which showed 2.888 was the threshold for NEAT1 (compared with GAPDH). Exactly mRNA levels of NEAT1 >2.888 was considered as “high” and those ≤2.888 as “low” (Figure 2(j), AUC = 0.9070). In our result, higher expression level of NEAT1 displayed the lower overall survival time (Figure 2(h), *p*=0.0012) and progression-free survival time (Figure 2(i), *p*=0.0064). We also found high expression of NEAT1 mRNA significantly correlated with distant metastasis (*p* < 0.01), lymph node metastasis (*P*=0.0001), and tumor grade (*P*=0.011) (Table 1).

### 3.2. Upregulation of NEAT1 in CRC Tissues and Cells

**Figure 3:**
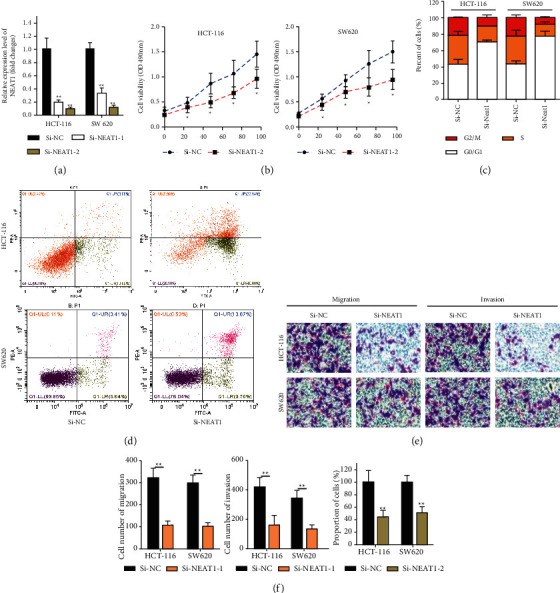
Suppression of NEAT1 in repressing cell proliferation and invasion of CRC. (a) SW620 and HCT-116 cells transfected with NEAT1-1 or NEAT1-2 for validation by qRT-PCR. (b) CCK-8 assay was prepared to detect cell proliferation of CRC. (c) Flow cytometry assay has explored the effect of NEAT1 knockdown in inhibition of cell cycle in SW620 and HCT-116 cells. (d) Flow cytometry assay was performed to discover NEAT1 knockdown in promotion of apoptosis in SW620 and HCT-116 cells. (e) Transwell assay was prepared to detect cell invasive and migration after silencing of NEAT1 expression in SW620 and HCT-116 cells. (f) Cell colony assay was used to verify silencing of NEAT1 expression decreases cell proliferation. ^*∗*^*P* < 0.05, ^*∗∗*^*p* < 0.01, compared with the NC group.

After transfection, the expression of NEAT1 significantly decreased in the siRNA NEAT1 group compared with the siRNA negative control group (Figure 3(a), *P* < 0.05). The CCK-8 assay showed that at 48, 72, and 96 hours, siRNA NEAT1 significantly repressed cell viability compared with the negative control group in SW620 (Figure 3(b), *P* < 0.05). We also detected NEAT1 knockdown inhibited cell proliferation by the colony formation assay (Figure 3(f)). Suppression of NEAT1 restrain much more cells than the negative control group in G0/*G*1 which inhibited cell proliferation (Figure 3(c), *P* < 0.05). Loss function of NEAT1 elevated the apoptotic rate in SW620 and HCT-116 by the flow cytometry apoptosis assay (Figure 3(d), *P* < 0.05). Afterwards, the transwell assay further indicated the silenced of NEAT1 decreased invasive and migratory cell numbers, which revealed NEAT1 led to CRC invasion and migration (Figure 3(e), *P* < 0.05).

### 3.3. Suppression of NEAT1 in Repressing Cell Proliferation and Invasion of CRC

**Figure 4:**
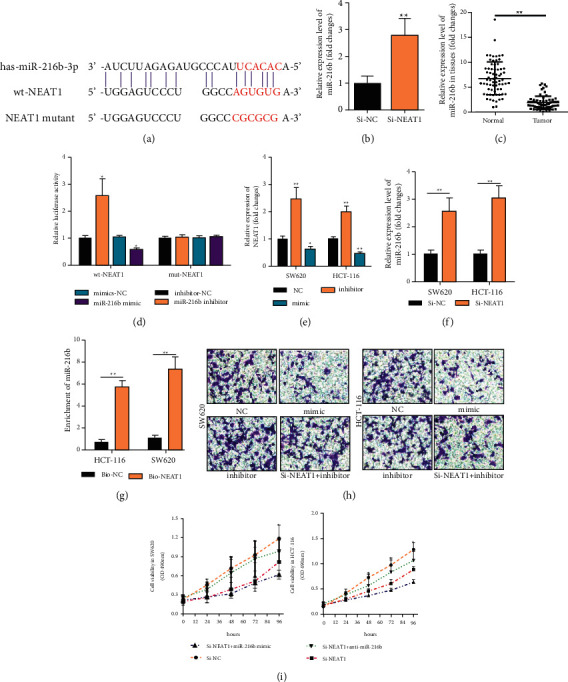
NEAT1-inactivating miR-216b to promote cell progression in CRC. (a) Online bioinformatics tool to analyze the binding site of NEAT1 and miR-216b. (b) Detecting expression of miR-216b mRNA expression by qRT-PCR in HCT-116 cell with NEAT1 silenced or control. (c) Exploring miR-216b expression in CRC tissues and normal tissues by qRT-PCR. (d) Detection of luciferase activity in cell lysates in HCT-116 cells cotransfected with wild-type or mutant NEAT1 plasmid and miR-216b mimic or miR-216b inhibitor. (e) Determining NEAT1 expression in HCT-116 cell transfected with miR-216b mimic or miR-216b inhibitor. (f) Discovering miR-216b expression in HCT-116 cell transfected with NEAT1 silence or control. (g) Detection of miR-216b by qRT-PCR in the sample pulled down by the biotinylated NEAT1 probe. (h) Exploration of cell migration and invasion by transwell assays in HCT-116 and SW620 cells transfected with miR-216b mimic, or the effect of miR-216b on proliferation in HCT-116 and SW620 cells cotransfected with NEAT1-silencing plasmid and miR-216b mimic or miR-216b inhibitor via CCK-8 assay. ^*∗*^*P* < 0.05, ^*∗∗*^*p* < 0.01, compared with the NC group.

We identified miR-216 b as a most potential candidate by bioinformatics database “Diana Tools” (Diana, http:// diana.imis.athena-innovation.gr/DianaTools) (Figure 4(a)). Since then, we constructed plasmids of NEAT1 to detect whether the decreased expression of NEAT1 could lead to the increased expression of miR-216 b as well as designed the luciferase reporter containing exact or mutant miR-216 b binding sites to determine the binding effect between NEAT1 and miR-216 b. We found NEAT1 knockdown resulted in the increased expression of miR-216 b by qPCR (Figure 4(b)). Furthermore, we found the decreased miR-216 b expression in the CRC tissue (Figure 4(c), *P* < 0.05). The luciferase reporter showed miR-216 b mimic significantly reduced luciferase activity of the wild-type NEAT1 plasmid, however, miR-216b inhibitor significantly increased it in HCT-116 ell line (Figure 4(d), *P* < 0.05). Moreover, miR-216b inhibitor significantly upregulated the expression of NEAT1(Figure 4(e), *P* < 0.05). In contrast, knockdown of NEAT1 also induced the upregulation of miR-216b (Figure 4(f), *P* < 0.05). Subsequently, the biotin-labeled pulldown system was applied to further confirm whether NEAT1 could pulldown miR-216 b. We observed miR-216 b in CRC cells pulled down by biotinylated NEAT1 which indicated miR-216b could directly bind to NEAT1 at the microRNA recognition site (Figure 4(g), *P* < 0.05). The transwell assay was prepared to explore the function of NEAT1 and miR-216b in migration and invasion, the result from which identified SW620 and HCT-116 transfected with NEAT1-silenced and cotransfected with miR-216b mimic significantly repressed the cell invasion and migration but failed when cotransfected with the miR-206 inhibitor (Figure 4(h), *p* < 0.01). The SW620 and HCT-116 cell lines with NEAT1 silence showed poor ability in proliferation and it achieved better when cotransfected with miR-216b mimic. However, when cell lines with NEAT1-silenced and cotransfected with miR-216b inhibitor displayed better ability in proliferation than that with NEAT1-silenced and cotransfected with miR-216b mimic(Figure 4(i), *p* < 0.01).

### 3.4. NEAT1 Sponged miR-216b to Activate YY1 to Regulate the Progression of CRC Cells

**Figure 5:**
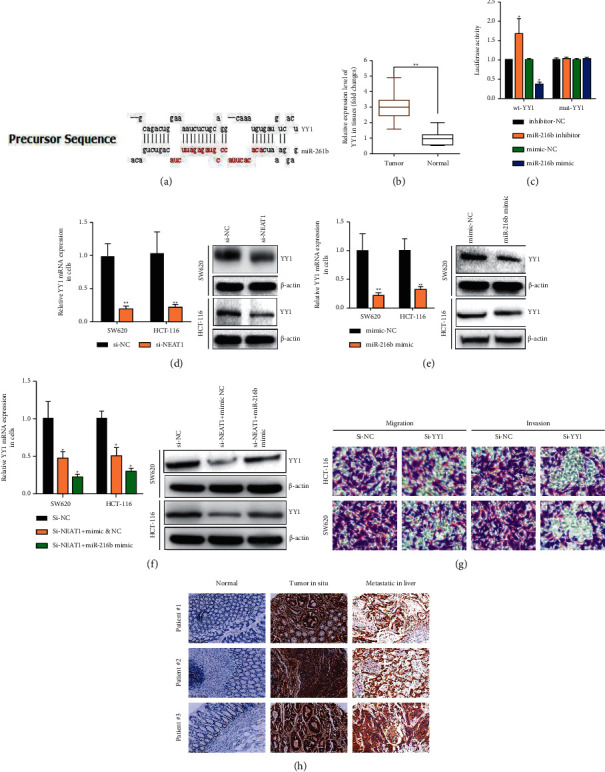
YY1 targeted by miR-216b and positively regulated by NEAT1 in CRC cells. (a) The 3′-UTR of YY1 contained miR-216b binding site by miRDB tool. (b) YY1 overexpressed in CRC tissues compared with normal tissues. (c) The luciferase activity was measured in HCT-116 cells transfected with miR-216b mimics or miR-216b inhibitor for 48 h. (d) The expression of YY1 mRNA and protein were detected by qRT-PCR and western blot assays in HCT-116 and SW620 cells with NEAT1 silence. (e) The expression of YY1 mRNA and protein were detected by qRT-PCR and western blot assays in HCT-116 and SW620 cells transfected with miR-216b mimic. (f) The expression of YY1 mRNA and protein were detected by qRT-PCR and western blot assays in HCT-116 and SW620 cells transfected with NEAT1 silence or cotransfected with miR-216b mimic. (g) Cell invasion and migration were tested by the transwell assay in HCT-116 and SW620 cells with NEAT1 silence. (h) The expression of YY1 protein in CRC tissues, liver metastatic tissues, and normal tissues was verified by IHC. ^*∗*^*P* < 0.05, ^*∗∗*^*p* < 0.01, compared with the NC group.

To explore candidate target of miR-216b, bioinformatics online tools (miRDB, http://mirdb.org/) were prepared to predict the suitable one that finding miR-216b possessed the matched binding site with YY1 (Figure 5(a)), which overexpressed in the CRC tissues not normal tissues (Figure 5(b), *p* < 0.01). In addition, the IHC staining indicated the strong staining of YY1 in the CRC tissues and liver metastatic tissues compared with the almost no staining in the CRC situ tissues especially in the liver metastasis tissues (Figure 5(h)), which suggested the oncogenic role of YY1 in CRC. It also showed the decreased mRNA and protein expression of YY1 in SW620 and HCT-116 with NEAT1-silence or miR-216b mimic (Figures5(c), 5(d), and 5(e), *p* < 0.01), nevertheless, it decreased much better when SW620 and HCT-116 with NEAT1-silence cotransfected with miR-216b mimic(Figure 5(f), *p* < 0.01). The cell migration and invasion ability in SW620 or HCT-116 was inhibited significantly when transfected with YY1 plasmid (Figure 5(g), *p* < 0.01). Taken together, NEAT1 sponged miR-216b to activate YY1 to accelerate the ability of cell viability, apoptosis, and invasion on colorectal cancer cells.

## 4. Discussion

NEAT1 participates in many critical biological processes and acts as a potential predictor or target for prognosis in CRC, lung cancer, gastric cancer, and breast cancer [9, 12, 19, 20]. However, further research studies are required to elucidate how NEAT1 function. Herein, we discovered high expression of NEAT1 in the CRC tissue, and associated with histological differentiation, overall survival, distant metastasis, and nodal metastasis, acting as an independent prognostic factor for overall survival in patients with CRC, indicating the poor prognosis. In the present study, we also observed that NEAT1 knockdown or miR-216b overexpression remarkably attenuated the ability of cell viability, apoptosis, and invasion on colorectal cancer cells. Mechanismly, our results disclosed that NEAT1 sponged the miR-216b to facilitate the function of the downstream regulators YY1, thus to promote CRC proliferation, invasion, and migration.

Accumulating evidence has indicated that LncRNA could regulate the expression of microRNA as ceRNA [3, 4, 21–23]. NEAT1 displayed multiple function in regulating microRNA that it not only sponged miR-133b to promote migration and invasion of breast cancer cells [24], but also inactivated miR-101 to play potential oncogene in breast cancer [25]. Moreover, in lung cancer, NEAT1 competed against let-7a to contribute to nonsmall-cell lung cancer proliferation and metastasis [26], and another research in lung cancer also revealed downregulation of NEAT1 led to cell invasion in NSCLC via sponging miR-153-3p [27]. In CRC, it was illustrated that the NEAT1 expression level was an independent prognostic factor for disease-free survival and overall survival which mechanismly promoted colorectal cancer progression by competitively binding miR-34a [12] resulting in activation of Wnt/beta-catenin signaling pathway. What's more, NEAT1 also could facilitate the sensitivity of 5-FU in CRC cells via miR-150-5p [14]. Although a little study displayed the anti-cancer role of NEAT1 in CRC, a flood of investigations have still been verified its oncogenic role especially acting as ceRNA to regulate microRNAs [20, 27]. However, Xiong's team revealed that the NEAT1 expression in the CRC tissues were not significantly different compared with the normal tissues [28]. Therefore, the function of NEAT1 in CRC needs to be further explored.

Like previous studies, our research also identified NEAT1 acting as oncogene in CRC and considered as an independent prognostic factor for disease-free survival and overall survival. With the help of online bioinformatics tools “DIANA” and “MIRDB”, we have explored microRNA-216b that NEAT1 may sponge to discover the deep mechanism. MicroRNA-216 b from our result was first reported molecular which can be regulated by NEAT1. MicroRNA-216 b was considered as the tumor suppressor in lung cancer, gastric cancer, and pancreatic cancer which could regulate cell proliferation, apoptosis, and invasion [29, 30]. Here, we found that miR-216b, acting as a connecting carrier, targets both NEAT1 and the 3′-UTR of YY1 by luciferase reporter assays. YY1 showed the oncogenic role in CRC, especially playing the epigenetic regulative role on cancer stem cell transcription factors to accelerate tumor metastasis [31, 32]. This constitutes the identification of a competitive endogenous RNA network in colorectal cancer. Although we verified NEAT1 sponged the miR-216b to facilitate the function of the downstream regulators YY1, our limitations should not be ignored in this study: our results were from experiments in vitro not in vivo, which made us to finish the further studies as well as the epigenetic regulation of YY1 in this axis.

## 5. Conclusion

Our results illustrate that NEAT1 functions as an oncogenic lncRNA to facilitate the carcinogenesis and progression of CRC by competitively sponging miR-216b to activate YY1. The present results elucidate a potential mechanism underlying the tumor-oncogenic role of NEAT1 in colorectal cancer and indicate that NEAT1 could serve as a useful marker and potential therapeutic target in CRC.

## Figures and Tables

**Table 1:** Correlations between NEAT1 and clinicopathological features of colorectal cancer patients.

Characteristics	Numbers	NEAT1 expression		*P*value
		High	Low	
Age (y)				
<60	26	15	11	0.83
≥60	31	17	14	

Gender				
Male	32	17	15	0.60
Female	25	15	10	

Size (cm)				
≤5	23	16	7	0.09
>5	34	16	18	

Histological differentiation				
Well	19	4	15	0.0002^*∗*^
Poor	38	28	10	

Nodal metastasis				
Positive	37	27	10	0.0005^*∗*^
Negative	20	5	15	

Distant metastasis				
Positive	12	11	1	0.005^*∗*^
Negative	45	21	24	

Grade				
I/II	19	6	13	0.0082^*∗*^
III/IV	38	26	12	

*p* value; ^*∗*^*P* < 0.05 and ^*∗∗*^*p* < 0.01.

**Table 2:** Univariate and multivariate analyses of clinicopathologic factors for survival in colorectal cancer patients.

Characteristics	Univariate analysis			Multivariate analysis		
	HR	95% CI	*P* value	HR	95% CI	*P* value
Age (≥60 vs. <60 y)	0.918	0.913∼3.175	0.101			
Gender (male vs. female)	0.786	0.870∼2.792	0.597			
Tumour size (≥5 vs. <5 cm)	0.866	0.365∼2.015	0.743			
Tumour grade (*T*1+T2 vs. *T*3+T4)	1.599	0.548∼6.224	0.344			
Histological differentiation (poor vs. well)	1.148	0.061∼2.706	0.135			
Nodal metastasis (N vs. P)	0.20	0.285∼0.519	0.001^*∗*^	6.69	3.537∼6.512	0.029^*∗*^
Distant metastasis (N vs. P)	2.667	2.537∼24.339	0.005^*∗*^	5.560	3.299∼29.566	0.028^*∗*^
NEAT1 expression (H vs. L)	2.271	0.114∼0.562	0.007^*∗*^	1.625	0.038∼3.506	0.012^*∗*^

HR : hazard ratio; CI : confidence interval. ^*∗*^ represents the *P* values with significant differences. N : negative; P : positive; H : high; L : low.

## Data Availability

The data that support the findings of this study are available from the corresponding authors upon reasonable request.

## Supplementary Materials

Supplementary Materials Table S1: PCR primers used in this work. YY1 targeted by miR-216b and positively regulated by NEAT1 in CRC cells.
