# Supplementary material for: The lncRNA NEAT1 Inhibits miRNA-216b and Promotes Colorectal Cancer Progression by Indirectly Activating YY1
Source: J Oncol. 2022 Oct 10;2022:8130132. doi: 10.1155/2022/8130132 (PMC9576420; doi:10.1155/2022/8130132)
Supplement: Supplementary Materials — Table S1: PCR primers used in this work. YY1 targeted by miR-216b and positively regulated by NEAT1 in CRC cells. [file 8130132.f1.docx]

**Table S1. PCR primers used in this work..**

| Name | sequencing |
| --- | --- |
| NEAT1 | Forward: 5'- CTTCCTCCCTTTAACTTATCCATTCAC -3' |
|  | Reverse: 5'- CTCTTCCTCCACCATTACCAACAATAC -3' |
| miR-216b | Forward: 5'- GCCGCGCTAAAGTGCTTA-3' |
|  | Reverse: 5'- CACCAGGGTCCGAGGT-3' |
| U6 | Forward:5’- CTCGCTTCGGCAGCACA-3’ |
|  | Reverse: 5’- AACGCTTCACGAATTTGCGT-3’ |
| GAPDH | Forward: 5'-CACCCACTCCTCCACCTTTG-3' |
|  | Reverse: 5'-CCACCACCCTGTTGCTGTAG-3' |
|  | |
| YY1 Forward:5'- AGCAGAAGCAGGTGCAGATCAA-3'  Reverse:5'- CTGCCAGTTGTTTGGGATCT -3' | |

The full‐length NEAT1 (accession number: NR_028272) was amplified with the following primers: forward, 5′CTTCCTCCCTTTAACTTATCCATTCAC‐3′; reverse, 5′‐ CTCTTCCTCCACCAT TACCAACAATAC‐3′.
